# Surface Coverage Simulation and 3D Plotting of Main Process Parameters for Molybdenum and Vanadium Adsorption onto Ferrihydrite

**DOI:** 10.3390/nano12030304

**Published:** 2022-01-18

**Authors:** Loredana Brinza

**Affiliations:** Department of Exact and Natural Sciences, Institute of Interdisciplinary Research, “Alexandru Ioan Cuza” University of Iasi, 700107 Iasi, Romania; Loredana.brinza@uaic.ro

**Keywords:** ferrihydrite, adsorption, molybdenum and vanadium, uptake capacity, removal efficiency, practical vs. theoretical surface coverage, applications, implications

## Abstract

Ferrihydrite, FHY, was synthesized and characterized for morphology, mineralogy, surface area, hydrodynamic diameter and surface charge properties before molybdenum (Mo) and vanadium (V) adsorption. The potentiometric titration results showed first direct evidence that CO_2_ affects FHY surface sites at pH 6–9. Beside CO_2_, particles concentration may affect surface properties with an impact on adsorption performance. Additional new adsorption simulation results on theoretical surface coverage vs. experimental results obtained at varying particles concentration help theoreticians and experimentalists to better estimate and apply anion adsorption processes to real environments and suggest that simulation may not always be entirely reliable. Uptake capacities obtained experimentally, varying pH, particles and metals concentrations, were plotted to assess their synergetic effect and derive trends for future process optimization. Adsorption kinetics and isotherms were also considered. Experimentally derived values for maximum uptake capacities (0.43 and 1.20 mmol g^−1^, for Mo and V, respectively) and partitioning coefficients have applications, such as in making decisions for anions removal from wastewaters to achieve depollution efficiency or concentration required for effluents discharge and also implications in elements cycling from a geochemical perspective. In this work, the 3D plotting of the main adsorption process parameters obtained experimentally showed inter-correlations between significant process parameters that influence the adsorption process, and provides guidelines for its optimization and indicates that laboratory data can be transposed to real systems.

## 1. Introduction

Ferrihydrite (FHY) is a well-known poorly ordered iron oxyhydroxide that forms in different terrestrial environments mostly under neutral conditions (i.e., deep-sea hydrothermal systems, soils, rivers) but also acidic ones such as acid mine drainage [1,2]. The most important characteristics of ferrihydrite are its small size and extremely high surface area, which leads to its high affinity to adsorb different cations and anions that could be considered contaminants. After contaminants adsorption onto ferrihydrite nanoparticles, they can transport over a long distance, causing environmental pollution on a wide scale.

Adsorption is a process frequently used in wastewater treatment and depollution technologies, soil treatment procedures as well as occurring in natural systems with the impact of the geochemical cycling of elements. A good adsorbent, from a process engineer point of view, for environmental depollution purposes, has to feature the following characteristics: easily available/synthesis and non-expensive, environmentally friendly, distinct surface properties (i.e., surface area and charge properties), good selectivity for target pollutants and a high uptake capacity to provide superior pollutant removal efficiency from polluted waters and additionally to be potentially reusable in multiple adsorption cycles.

Of the anions that may constitute pollutants if their concentration in surface waters exceeds the limits imposed by environmental standards [3,4], the interactions between molybdenum (Mo) and vanadium (V) and ferrihydrite have so far been little studied [5,6,7,8,9,10,11]. Besides our previous studies [5,6,12], which present high resolution qualitative and quantitative experimental investigations of molybdenum and vanadium interactions with ferrihydrite, the literature contains mostly modelling/computational studies that can be used to simulate the transport and bioavailability of these anions in the environment [7,9,10,13]. From our experimental vs. modelling experience, we have shown that some discrepancies may exist between the two approaches. Thus, more experimental investigations are needed to understand factors that affect anion uptake under various experimental conditions, differences between practical and theoretical values, parameters and correction factors which can further be used in applications for wastewaters treatments or estimates of the geochemical cycling of elements in various settings. Among the factors that affect depollution performance, the pH is one of the main factors, as it affects the adsorption process via elements chemistry in solution as well as adsorbent surface properties. Besides pH, adsorbent dosage or particles concentration and elements concentration are key parameters that significantly impact adsorption performance. Thus, they will be investigated in the current work. The adsorption results are presented in various ways such as: uptake capacity, adsorption efficiency, Kd, etc. An important parameter in assessing the performance of wastewater treatment/depollution is pollutant-removal efficiency, which is indirectly related to the adsorption uptake capacity of the adsorbent used. The first expresses the percentage of the pollutant removed from the polluted water, which is a consequence of pollutant concentration and the dose of the adsorbent loaded for its treatment at specific environmental conditions, but it does not take into consideration the adsorbent properties in its calculation. The adsorption uptake capacity is a parameter that features the adsorbent and is an expression of the maximum pollutant loading onto an adsorbent surface. In the literature, the results are mainly expressed as maximum pollutants uptake capacity; however, a correlation among these parameters and factors that affects the adsorption is essential for a process engineer to obtain a clear view of process optimization and scaling up. Additionally, partition or distribution coefficient, Kd, is a highly important parameter for estimating the uptake processes of various pollutants in soil and sediments or in other geological matrices. It can be obtained experimentally and is used to computationally estimate, via modelling codes, the transport of contaminants and their risk assessment. The United States Environmental Protection Agency (U.S. EPA) has considered standard methodologies to experimentally determine Kd and also a compilation of Kd values for selected pollutants onto various substrates, compiled as a database from the literature [14]. Kd values may vary substantially, across many orders of magnitude, as a function of pH, ionic strengths (IS), redox and the presence of other constituents to a system (organic matter, sulfides, clay content, cation exchange capacity, dissolved Ca and Mg, etc). For example, Kd for Cd on soils at low IS, humic content and no organic chelates and under oxidizing conditions can take values from a minimum of 1-at pH 3–5, to a maximum of 12,600, at pH 8–10 [15]. However, in order to assess the synergetic effect of numerous constituents to a natural heterogeneous system, the need to investigate individual systems to derive values for simpler systems has arisen and is imposed. Based on these values, modelling codes can further derive interrelated values for Kd, as well as set limits concerning factors affecting it, and ultimately to provide a Kd value close to a particular system for which an estimate for pollutants transport or risk assessment is to be carried out. 

From the point of view of data expression modes, as seen above and in the literature, the adsorption data are also expressed by normalized uptake capacity per surface charge densities or surface area of the adsorbent rather than adsorbent mass [16]. An experimental study assisted by the computational extension of the As adsorption onto goethite covered sand suggested that adsorption normalized to surface area rather than to the adsorbent mass further reduces variability in Kd and q, both for pH-dependent and concentration-dependent adsorption [16]. The choice of expressing the adsorption data is mainly driven by aim of data applicability. 

In this study, ferrihydrite was synthetized and well characterized, with the aim to investigate the molybdenum and vanadium uptake via adsorption by varying the following main process parameters: pH (4 to 9); particles concentration (0.1 to 2 g L^−1^) and metals concentration (1 to 750 µmol L^−1^) conditions. The effect of CO_2_ on ferrihydrite surface charge was investigated for the first time in a direct manner by potentiometric titrations. Adsorption results are expressed in various ways to allow for a literature comparison to be conducted, provide values for future reference and for increase data applicability. Practical and theoretical adsorption surface coverage data and discussions were made to enhance existent differences and identify correction factors that need to be considered in future simulations. The experimental results obtained from the sets of experiments by varying the main process conditions were plotted in 3D to demonstrate inter-correlations between adsorption parameters for process optimization and to also provide guidelines for how laboratory data can be used to scale up and for transposition to real systems. Finally, brief examples of how the obtained results can have applications in wastewater depollution and implications to elements geochemical cycling are given.

## 2. Materials and Methods

### 2.1. Materials Synthesis and Characterization

All chemicals (HCl, NaOH, Fe(NO_3_)_3_ × 9H_2_O, Na_2_MoO_4_, Na_3_VO_4_) were of analytical grade and were purchased from Sigma Aldrich (Dorset, UK). Solutions were prepared using 18 mΩ cm Millipore deionized water.

Ferrihydrite was synthesized following Cornell and Schwertmann (2000)’s method [17], by neutralizing a ferrous nitrate solution (0.2 mol L^−1^) with NaOH (1 mol L^−1^) to pH 7, under vigorous stirring. After synthesis, the slurry was washed by successive centrifugation/dispersion cycles using double distilled water (DDW). After drying, the solids were characterized by X-ray Diffraction (XRD, Bruker D8, Billerica, MA, USA), Brunauer, Emmett, and Teller (BET, Micromeritics Gemini V, Dunstable, Bedfordshire, UK) analyser, Scanning Electron Microscopy (SEM, LEO 1500 Series, Cambridge, UK), and Transmission Electron Microscopy (TEM, FEI CM200, Eindhoven, The Netherlands), while the slurries were analyzed with potentiometric titrations (PT, Man-Tech Inc., Guelph, ON, Canada) and Dynamic Light Scattering (DLS, Malvern Mastersizer, Malvern, UK). The dry weight (DW) of the solids was determined after drying overnight in an oven at 40 °C. FEG SEM/TEM allowed for the evaluation of the size and morphology of individual particles and aggregates as well as the identification of the solid mineral phase composition and their crystallographic parameters. FEG–SEM imaging was performed with a LEO 1500 Series microscope with a GEMINI column. Images were collected at 3 keV at a working distance of 3–6 mm with the samples deposited on an Al stub and after coating with 3 nm of Platinum. TEM investigations were carried out with an FEI CM200 FEG-TEM operating at 197 kV which was fitted with a Gatan Imaging Filter (GIF 200). Selected area electron diffraction, (SAED), bright field images (BFI), and energy dispersive X-ray Spectrometric (EDS, Oxford Instruments) analyses were used to obtain quantitative diffraction patterns (SAED) and semi-quantitative molar ratios for Mo:Fe and V:Fe (EDS). In all cases, the EDS analyses were collected on at least 3 different points on a sample to assess the homogeneity of the element distribution in the samples.

Potentiometric titrati ons at a low ionic strength in the presence and the absence of CO_2_ were carried out using a Man-Tech auto-titration system equipped with an automatic burette in order to obtain the point of zero charges of the ferrihydrite. Dynamic light scattering measurements were carried out in water matrix to determine the size range of ferrihydrite aggregates in the liquid phase at different particle concentrations, pH 7 and in static regime. Further details about nanoparticles characterization and apparatus settings are presented in Supplementary Information (SI).

### 2.2. Adsorption Experiments

Adsorption experiments were carried out in batch experiments using wet ferrihydrite as a slurry with a known density, varying pH, metal, and also solid concentrations. The pH-dependent adsorption tests (4–9) were carried out under a well-controlled and monitored pH environment, ensured by a Mantech titrator. Batch experiments were conducted in 500 mL glass beakers, under N_2_ atmosphere, using 100 µmol L^−1^ molybdenum and vanadium solution and 0.1 g L^−1^ ferrihydrite. Further details are to be found in previous works [5,6]. A pH adsorption edge diagram as well as their kinetic profile plotting and modelling were treated in our previous work [6]. Here, quantitative values at equilibrium were only taken for the 3D plotting of main adsorption parameters and to derive information about their interrelations.

Adsorption experiments run under varying particles concentration (from 0.1 to 2 g L^−1^, at metal concentration of 100 μmol L^−1^) and metals concentrations (from 1 to 750 µmol L^−1^, at particles concentration of 0.1 g L^−1^) were conducted at pH 7 at a mixing speed of 300 rpm (rotations per minute) and room temperature (23 ± 2 °C). During the adsorption supernatant solution, samples were collected following a geometrical time scale of 0–1280 min, filtered (using 0.2 μm Cellulose Acetate Filters) and prepared for molybdenum and vanadium analyses. Molybdenum and vanadium concentrations were measured using a Perkin Elmer Optima ICP-OES with a detection limit of 0.6 μg L^−1^ and 0.9 μg L^−1^, respectively. All experiments were run in triplicate to meet the requirements of statistically relevant number of samples, and standard deviation was calculated below 5%. 

The metal uptake was calculated from the system mass balance using Equation (1) [18].

(1) $$q_{}=\frac{V(C_{i}-C_{e})}{m}$$

The data were also expressed as the efficiency of metal removal from the solution using Equation (2).

(2) $$E=\frac{\left(C_{i}-C_{e}\right)}{C_{i}}\cdot 100$$

where *Ci* is the initial concentration of the metal in the solution, *Ce* is the concentration of the metal at equilibrium.

The kinetic results obtained from the experimental set in which particles concentration was varied were fitted with a pseudo-first order kinetic model (PFO) [19] and pseudo-second order kinetic model (PSO) [20,21] to derive the weighted values for the uptake capacities at equilibrium under specified conditions. These values were used in further 3D plotting and to obtain empiric information about adsorption mechanisms. Details about the kinetic models used are to be found in the SI. Best fits were evaluated as highest adjusted R^2^ value and the lowest chi-square.

The results from the effect of metal concentration were plotted as adsorption isotherms (q_e_ (the amount of metal adsorbed at equilibrium) vs. Ce (the concentration of the metal at equilibrium)) and fitted with the Langmuir model (Equation (3)) and Freundlich model [22,23,24,25], to allow for the calculation of weighted maximum uptake capacities that will be used for (i) literature comparison with other adsorbents; (ii) intercorrelation of adsorption process parameters via 3 D plotting and simulated examples of real environmental applications/implications. The maximum concentration of anions was selected to be below the anion saturation in solution. According to our previous solution chemistry modelling results (see SI of Brinza et al., 2019 [6]), no molybdenum and vanadium polymerization or precipitation phases were expected to occur at chosen anions concentration. The lack of Mmolybdenum and vanadium precipitated or polymerized phases at ferrihydrite surface were also proved by our previous XAS results [6]. 

The Langmuir model is a theoretical model that assumes adsorption sites are homogeneously distributed on the adsorbent surface, are energetically similar, and that the adsorbate adsorption takes place as monolayers. It accounts for the surface coverage by balancing the relative rates of adsorption and desorption (dynamic equilibrium). Adsorption is proportional to the fraction of the surface of the adsorbent that is open while desorption is proportional to the fraction of the adsorbent surface that is covered.

(3) $$q_{max}=\frac{q_{e}bC_{e}}{1+bC_{e}}$$

*q_max_* is the maximum adsorption capacity, (mmol g^−1^), *Ce* is metal concentration in solution at equilibrium (mmol L^−1^) and b is dimensionless Langmuir constant related to the feasibility of adsorption.

Derived from the Langmuir isotherms, it is a so-called separation factor R_L,_ which can be calculated by the following equation.

(4) $$R_{L}=1/\left(1+bC_{o}\right)$$

*b* is the Langmuir constant and *C_o_* is adsorbate initial concentration (mmol L^−1^).

The values of *R_L_* assumes the nature and the feasibility of the adsorption process. *R_L_* values above 1 indicate an unfavorable adsorption process, *R_L_* = 1 indicates a linear adsorption process, a 0 < *R_L_* < 1 indicates that the absorption process is favourable and for *R_L_* = 0 the adsorption process is irreversible [26].

The Freundlich isotherm is an empiric model (Equation (5)) that assumes the adsorption sites are heterogeneously distributed, and that adsorption takes place as multilayer. It provides an expression that defines the surface heterogeneity and the exponential distribution of active sites and their energies ([27]).

(5) $$q_{e}=K_{f}C_{e}{}^{\frac{1}{n}}$$

*qe* is the adsorption capacity at equilibrium, (mmol g^−1^), *Ce* is metal concentration in solution at equilibrium (mM), *Kf* is Freundlich constant (mmol g^−1^), 1/n is Freundlich exponent related to the adsorption intensity, and it also indicates the relative distribution of the energy and the heterogeneity of the adsorbate sites.

Briefly, both models are theoretical and/or empirical expression of adsorption equilibrium, and they involve various assumptions used to derive indirect mechanistic information, but they can also be used to obtain and compare adsorption capacities of various adsorbents for specific adsorbates or of various adsorbates for the same adsorbent, under similar or, ideally, identical process conditions.

### 2.3. Calculation of the Surface Sites Coverage

A calculation of the available surface sites and theoretical surface coverage of molybdenum and vanadium on ferrihydrite was performed using Equation (6), as follows:

(6) $$\Gamma (mol\;sites/L)=\frac{N_{S}(sites/m^{2})\times S_{A}(m^{2}/g)\times C_{S}(g/L)}{N_{A}(sites/mol\;sites)}$$

where Γ is the concentration of surface sites, *S_A_* is surface area = 200 m^2^ g^−1^ (from BET measurement), *N_S_* is surface site density = 2.27 sites/nm^2^ [28] and *C_S_* is solid/liquid ratio.

### 2.4. 3D Plotting of the Adsorption Parameters

The relevant adsorption process parameters of process engineering (i.e., pH, metal concentration, particles concentration) were plotted in 3D using the Origin 8 software (OriginLab, Northampton, MA, USA) [29] to demonstrate the interdependency between chosen parameters and to derive information about further process optimization. Parameters intercorrelation plots were carried out using the Kriging model [30,31,32,33].

## 3. Results

### 3.1. Ferrihydrite Characterization

#### 3.1.1. High-Resolution Microscopy and X-ray Diffraction

**Figure 1:**
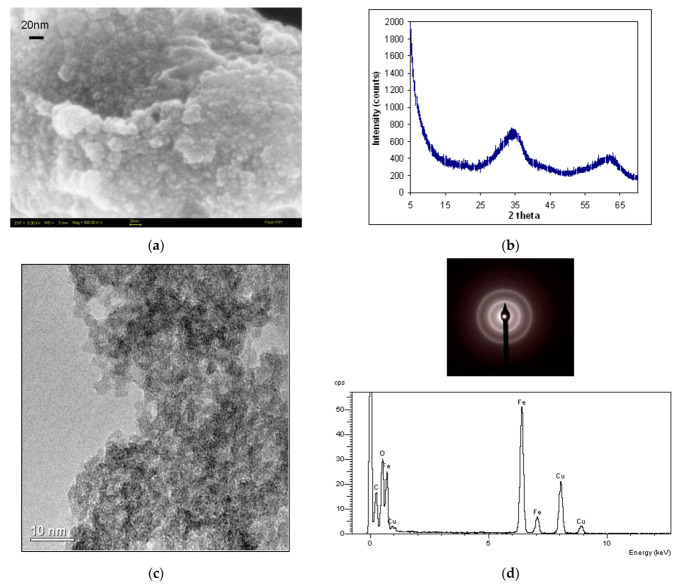
(**a**) FEG-SEM micrograph of the ferrihydrite shows small (≤5 nm) particles forming big aggregates. Scale bar is 20 nm; (**b**) XRD spectrum of 2-line ferrihydrite; (**c**) FEG-TEM micrograph of the ferrihydrite aggregate showing individual particles forming clusters and (**d**) above is Selected Area Electron Diffraction (SAED) and below is the Energy Dispersive Spectra (EDS) of the TEM imaged sample.

FEG-SEM and FEG-TEM images of the synthetized solid (Figure 1a,c) show aggregates of nanoparticles with a size of less than 20 nm. The XRD mineralogical identification (Figure 1b) show classical broad peaks at ca. 33° and 62° 2-theta corresponding to 1.5 Å and 2.6 Å d-spacing, respectively, which are representative of highly amorphous 2-line ferrihydrite and confirm the expected mineralogical phase with no impurities.

The SAED spectra shows the diffuse diffraction rings characteristics of ferrihydrite (inset Figure 1d above), which could be assigned to d-values of 2.5 Å and 1.5 Å that are typical XRD diffraction peak positions for 2 –line ferrihydrite. The EDS spectra show Fe and O as the main constituents of ferrihydrite, while visible Cu and C peaks are indicative of the Cu grid that the TEM stub is made of, and the C is used for increasing sample conductivity. The SAED results support the XRD mineralogical and crystallographic results and confirm the identity of ferrihydrite.

#### 3.1.2. Dynamic Light Scattering and-Particle Size Measurement

Dynamic light scattering measurements were conducted to determine the size range of ferrihydrite aggregates in the liquid phase at different particle concentrations, pH 7 and in static regime.

**Figure 2:**
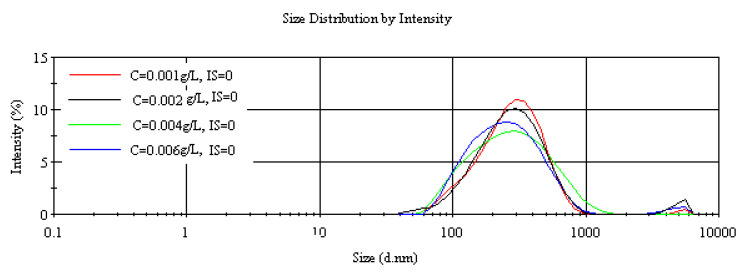
Particles size distribution at ionic strength 0 for four different particle concentrations (Average of 3 measurements, 10 runs per measurement).

The results (Figure 2) show that for particle concentrations of 0.001, 0.002, 0.004, and 0.006 g L^−1^, average sizes of the aggregates at zero ionic strength were 261, 252, 226, and 221 nm, respectively, indicating a small decrease in aggregate size with increasing particles concentration in the system. This effect can be explained by the effect of the Brownian motion in the system, which enhances particle dispersion and separation. From the shape of the plots (their broadness), it can be noted that a decrease in particles concentration led to slightly more monodisperse particles (particle distributions curve narrows at small particle concentration).

#### 3.1.3. Potentiometric Titration–Surface Charge Characterization

The influence of CO_2_ on the ferrihydrite surface properties is evident from the volume of base added to reach the same pH value and the shape of the titration curve.

**Figure 3:**
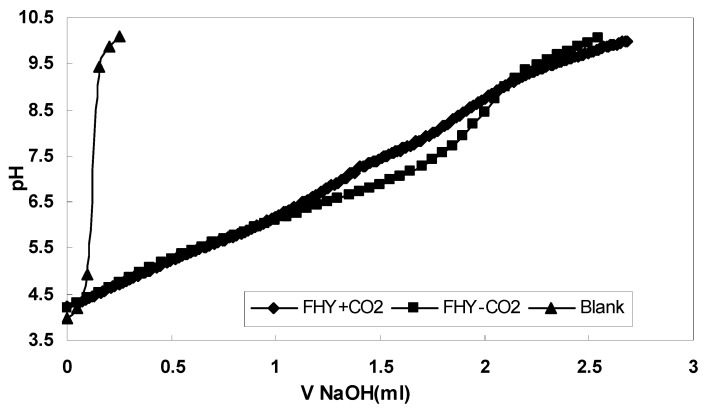
Potentiometric titration results for ferrihydrite surface charge characterization: the effect of CO_2_ onto ferrihydrite surface charge.

The first derivative of the pH/V plots provides the pH value that corresponds to the endpoint (ep) and thus the pH value for which the number of positive charges is equal to the number of negative charges (Figure 3). The potentiometric titration results presented here confirmed that the pH at point of zero charges of ferrihydrite was 7.96, which is in agreement with previous studies that found values of 8.1 and 8.2 [34,35,36,37].

In addition, when the ferrihydrite slurries were titrated in the presence or absence of CO_2,_ the data showed that CO_2_ influences the ferrihydrite surface properties between pH 6 and 9. These are the first experimental data from which direct observations on carbonate sorption onto ferrihydrite can be made. The second derivative of the pH/V gives acid dissociation constants for functional groups at the adsorbent surface, which reveal the deprotonation state of a molecule in a particular solvent. The pKa value found is 6.23 ± 0.05, and can be assigned to a cis isomer of –CO_2_H [38,39,40].

#### 3.1.4. BET-Surface Area Measurements

BET measurements of starting ferrihydrite provided a surface area of 198 (± 2.18) m^2^ g^−1^. In the literature, surface area values for ferrihydrite vary between 133 and 700 m^2^ g^−1^ [1,2,35,41,42,43]. Thus, our experimental results are in good agreement with those found in the literature. In surface complexation modelling, a theoretical value of 600 m^2^ g^−1^ is usually used [34,36,37,44]. For further simulation of the practical surface coverage, a value of 200 m^2^ g^−1^ will be considered.

### 3.2. Adsorption Results

#### 3.2.1. Particle Concentration Effect

**Figure 4:**
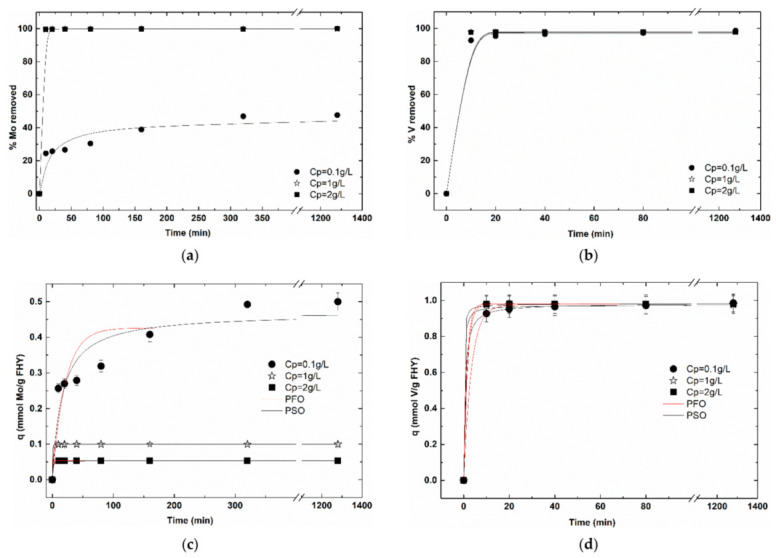
The kinetics of molybdenum (**a**,**c**) and vanadium (**b**,**d**) adsorption onto ferrihydrite at different particle concentrations (C_Mo/V_ = 100 μmol L^−1^; C_FHY_ = 0.1–2 g L^−1^, T = 23 ± 2 °C, pH 7) expressed as: (**a**,**b**) removal efficiency and (**c**,**d**) uptake capacities. Dots are experimental data, and lines are fitted data with a pseudo-first order (PFO) kinetic and pseudo-second-order (PSO) kinetic models.

The results from the experiments with variable particle concentrations (Figure 4a–d) indicate that for the molybdenum system at concentrations of above 1 g L^−1^, all molybdenum was totally removed from the solution in a very short time frame (ca. 5 min). At a particle concentration of 0.1 g L^−1,^ however, only 40% of molybdenum was adsorbed (ferrihydrite binding sites being saturated) and the adsorption equilibrium was achieved after 300 min. Conversely, for the vanadium systems, under all three conditions, 100% of vanadium was removed after 5–10 min of contact time. These results underline the higher affinity of vanadium compared to molybdenum for ferrihydrite surfaces at pH 7. For the removal efficiency of the molybdenum system, data show that at particle concentrations of 0.1 g L^−1^, ferrihydrite binding sites become saturated at 40% of the total 100 μmol L^−1^ molybdenum, whereas at the same particle concentration of all vanadium was removed from the solution. The 100% removal efficiency of 100 μmol L^−1^ molybdenum at particles concentration above 1 g L^−1^, indicates an excellent performance of adsorption process. For the vanadium system at all working particle concentrations, the vanadium was totally removed (Figure 4b).

Figure 4c shows that, at lower particle concentrations, molybdenum is adsorbed onto the ferrihydrite surface with a maximum capacity of 0.44 mmol g^−1^, within 320 min. As the particle concentration increases to 1 g L^−1^ and 2 g L^−1^, respectively, less molybdenum per g ferrihydrite (0.14 mmol g^−1^ and 0.08 mmol g^−1^) was taken up, and this process occurs much faster, with equilibrium reached within first 20 min. This trend can be explained by considering the distribution of a limited amount of molybdate ions (100 μmol L^−1^) onto an increasing number of available binding sites (high particle concentration). For the vanadium systems, high uptake capacities (ca. 1 mmol g^−1^) were reached at all particles concentrations (Figure 4d) in very short time frame (i.e., 10–20 min). In other words, as the particle concentration increases between 0.1 to 2 g L^−1^, ferrihydrite can adsorb progressively constant amount of vanadium from solution up to a specific value at which ferrihydrite reaches its saturation. A summary of the molybdenum and vanadium practical uptake capacities at equilibrium and kinetic rates (q_e_ and k_1_ and k_2_) as well as statistical relevant fitting parameters (red chi square and Adj. R^2^), as obtained from the pseudo-first and pseudo-second-order kinetic models fits, are given in Table S1. Kinetic fitting results for both, molybdenum and vanadium, show better fits by the pseudo-second order kinetic model for particles concentration of 0.1 g L^−1^ (Adj R^2^ = 0.883 for molybdenum and Adj R^2^ = 0.999 for vanadium) and by the pseudo first order kinetic model at increasing particles concentrations 1 g L^−1^ and 2 g L^−1^ (Adj R^2^ = 1 and Adj R^2^ = 0.999, respectively, for molybdenum and Adj R^2^ = 0.999 and Adj R^2^ = 1, respectively, for vanadium). Although empiric, these results suggest that the uptake mechanism changes with increasing particles concentration, from a predominantly chemical sorption at low particles concentration to a predominantly physical sorption at higher particles concentrations.

Below, the q_e_ weighed values from the best kinetic model fits, labelled as q_pr_ are presented for comparison with calculated theoretic values.

Empirically, if we consider a ferrihydrite surface area of 200 m^2^ g^−1^, a value for the ferrihydrite site density of 2.27 sites/nm^2^ [28,45] and metal concentration of 100 µmol L^−1^, the surface sites available for adsorption can be calculated by Equation (6) and the results are displayed in Table 1. Theoretically, a particle concentration of 0.1 g L^−1^, 100 µmol L^−1^, of molybdenum or vanadium, should cover all ferrihydrite surface sites (available 75.4 µmol L^−1^) to saturation, and ca. 25% of the available molybdenum and vanadium ions still should be free in solution. Additionally, at 1 g L^−1^ and 2 g L^−1^, only 13% and 7%, respectively, of the surface should be covered by the 100 μmol L^−1^ of mo-lybdenum and vanadium anions. From the above, a comparison of theoretical vs. practical E% and q can be made to evaluate simulation vs. experimental approaches and derive additional information about surface saturation, as detailed below.

In practice, for molybdenum at 0.1 g L^−1^, from the E% point of view, 60% of the molybdenum remains in the solution and not 25% as theoretically calculated (or 40% practically adsorbed vs. 75% theoretically adsorbed). From the maximum uptake capacity point of view, a q of 0.47 mmol Mo g^−1^ was obtained rather than a maximum of 0.75 mmol Mo g^−1^ as theoretically calculated (from sites density of the 0.1 g FHY and 100 µmol L^−1^ molybdenum available in the system = 0.75 mmol sites g^−1^ occupied fully by 0.1 mmol L^−1^ Mo), see Table 1. From the practical vs. theoretical comparison, it is noticeable that for both parameters E% and q, the trends are similar; however, better adsorption performances are calculated when compared to experimentally derived values. For the molybdenum systems at increasing particles concentration (i.e., 1 and 2 g L^−1^), the theoretic efficiencies and uptake capacities values (calculated accounting on the theoretical values for surface coverage) are in good agreement with both of the practical values obtained experimentally, suggesting that, under these conditions, computing results are a good representation of the real system. For the vanadium system at 0.1 g L^−1^, both, the removal efficiencies as well as the uptake capacities, practical values, are 25% higher than theoretical ones. As particles concentration increases, theoretic values for vanadium removal are similar to the practical ones, while, however, the theoretical values for the vanadium uptake capacities are substantially lower than the practical ones. 

To explain differences in practical vs. theoretical E and q, for molybdenum and vanadium it may need to carefully consider aspects such as the anions chemistry in solution (different deprotonation stages) and/or potentially different sorption mechanisms (monolayer vs. double-layered adsorption that might occur) and or a combination of adsorbent parameters chosen in the theoretical calculation, i.e., surface site densities and surface area, as well as the effect of particles concentration on particles size, as suggested from our DLS measurements. Generally, it is possible that some theoretical vs. practical discrepancies for both molybdenum and vanadium, mainly referred to as q (which accounts for adsorbent properties), may be attributed to the theoretical value chosen for surface sites density, which might not be representative of the systems under all conditions studied in practice. Thus, as a solution here, after a sensible identification of the cause, a reverse calculation can be suggested to obtain a new real value. This is beyond the scope of this paper and can act as the subject of future work. On the other hand, practical vs. theoretical quantitative discrepancies of q might be attributed to potential differences of ferrihydrite surface area values in solid-state vs. solution. Although our modelling used the value obtained from the current measurement (which was performed in a dry state), this value might be slightly altered for the ferrihydrite in solution. 

Kd values that were obtained experimentally for vanadium and molybdenum adsorption onto ferrihydrite take values from 444 to 749 and 2 to 2983, respectively. For the vanadium system, the Kd increases with decreasing ferrihydrite concentrations, and all values are of the same order of magnitude. Conversely, for the molybdenum system, huge discrepancies among values obtained from the experiments at various particle concentrations were observed for Kd, and no coherent trend was observed.

#### 3.2.2. Metals Concentration Effect and Adsorption Isotherms

**Figure 5:**
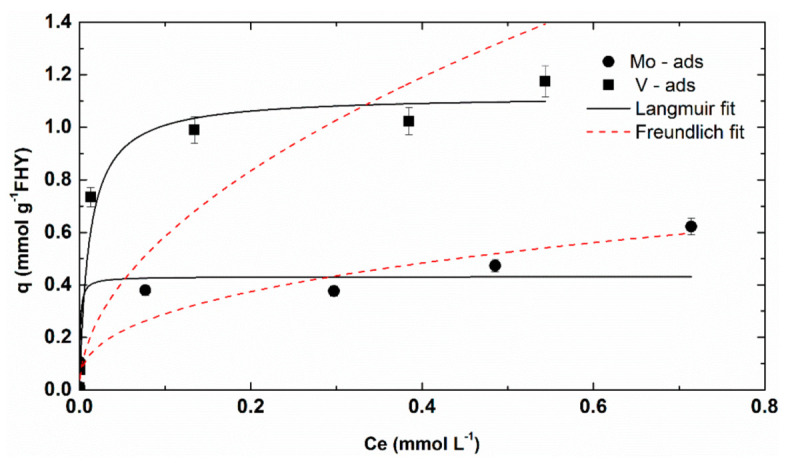
Adsorption isotherms of molybdenum and vanadium at pH 7, Ionic strengths (IS) 0.01, C_FHY_ = 1 g L^−1^, C_Mo/V_= 1–750 μmol L^−1^, and associate fitting parameters.

Adsorption isotherms obtained from the set of adsorption experiments, for which metal concentration varied from 1–750 μmol L^−1^, are presented in Figure 5 and associated parameters are to be found in Table S2.

The adsorption results showed that the maximum uptake capacities for molybdenum and vanadium via adsorption are 0.43 mmol g^−1^ and 1.28 mmol g^−1^, respectively. Better fits were observed to the Langmuir isotherm compared to the Freundlich isotherm for the molybdenum as well as vanadium (Supplementary Table S2), which empirically suggests that adsorption occurs in the monolayer. 

RL results calculated for molybdenum and vanadium at various initial concentrations showed positive values (from 0.003 to 0.72 for vanadium and 0.990 to 1 for molybdenum), suggesting that the adsorption processes are favourable (for 0 < R_L_ < 1) and linear (for R_L_ = 1).

The Kd values calculated for the current experiments at different anions concentrations have a decreasing trend with increasing anion concentration, RL and q values, for both vanadium and molybdenum (Table 2). It varies with an increasing anion concentration within three orders of magnitude, from 2.1 to 236.77, for vanadium and within four orders of magnitude, from 0.87 to 539.68, for molybdenum.

### 3.3. 3D Plotting of the Adsorption Parameters

The 3D plotting of the adsorption parameters varied in the experimental studies aims to elucidate the inter-correlations between crucial parameters that affect the adsorption process and to provide guidelines for how laboratory data can be used to scale up and transpose the experimental data to real systems and potentially aid at process optimization from an process engineering point of view if process will be implemented in depollution/remediation technologies at wastewater/sediments treatment sites.

**Figure 6:**
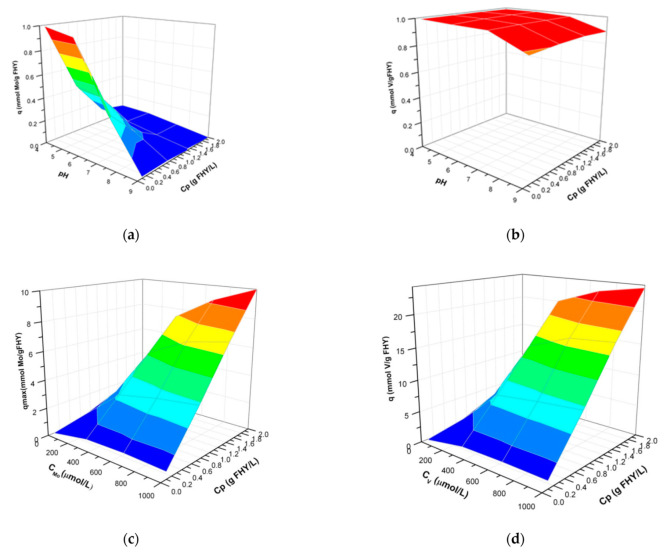
The profile of molybdenum (**a**) and vanadium (**b**) adsorption uptake capacities onto ferrihydrite at a metal concentration of 100 µmol L^−1^, as a function of pH and ferrihydrite particle concentration (Cp) at ionic strength ~0.01. The profile of molybdenum (**c**) and vanadium (**d**) adsorption uptake capacities onto ferrihydrite at pH 7, as a function of metal concentration (C_Mo_ and C_V_, respectively) and ferrihydrite particle concentration (Cp) at ionic strength ~0.01.

In Figure 6a,b, the molybdenum and vanadium uptake capacities, derived from the experimental data (IS = 0.01 and C_Mo/V_ = 100 µmol L^−1^) are plotted as a function of pH and particles concentration (Cp). For the molybdenum system, the uptake capacity decreases with an increasing pH (4 to 10) and increasing particle concentration (from 0.1 to 2 g L^−1^). However, considering that the total amount of molybdenum to be adsorbed was 100 µmol L^−1^ (the initial concentration of molybdenum), the decreasing trend of the uptake capacity with increasing particles concentration can be explained by the limited amount of molybdenum available for adsorption with the increasing number of sites. For the vanadium system (Figure 6b), the decrease in uptake capacity with increasing pH and particle concentration follows a comparable trend to the molybdenum system. Vanadium has a higher affinity for the ferrihydrite surface over a larger pH interval, a trend that can also be observed in the 3D diagrams.

The 3D plotting of the variation of metal concentration, particles concentration, and uptake capacities of molybdenum (Figure 6c) and vanadium (Figure 6d) shows that both systems follow the same trend and shape. However, the profile of vanadium differs from the molybdenum profile, reaching more than twice the uptake of molybdenum (q axis) over the same metal and particle concentration intervals. Thus, over the interval of 0–750 µmol L^−1^, with increasing particles concentration to 2 g L^−1^, the maximum uptake capacity of the ferrihydrite at pH 7 was found to be 10 mmol g^−1^ and 23.6 mmol g^−1^, for molybdenum and vanadium, respectively.

## 4. Discussion

### 4.1. Ferrihydrite Characterization

The microscopic investigations showed that the ferrihydrite formed aggregates made of poorly ordered ferrihydrite nanoparticles (ca. 5 nm in size), results that are in accordance with other literature findings [41,43,46,47,48,49].

The aggregates’ hydrodynamic diameter measured in dynamic regime by DLS showed a slight decrease in aggregate size and polydispersity with increasing particles concentration. This trend may be explained by an increasing Brownian motion in the system, which led to particles separation and dispersion, hence smaller particles size. Smaller particles with increasing particles concentration will have a positive impact on the performance of adsorption of different elements. The first trial of measuring the average diameter of ferrihydrite aggregates was carried out by Scheinost et al. (2001), who obtained a value of 30 µm. In addition, they also found that the aggregates of freeze-dried ferrihydrite were smaller with a mean diameter of 15 µm [50]. More recently, DLS measurements of ferrihydrite colloids (iron concentration ~1.4 mol L^−1^) and bulk (iron concentration ~9.6 mol L^−1^) investigated by Bosch et al., gave a value for colloidal ferrihydrite size of 336 ± 40 nm. Their results, for the particle concentration range of 0.001–0.005 g L^−1^, agree very well with our DLS measurement [51]. However, our data shows two orders of magnitude difference between the particles sizes measured by the DLS vs. the Mastersizer measurements (252 nm vs. 30 µm) for the suspension at an ionic strength of 0 and particle concentration of 0.002 g L^−1^ [6]. As these techniques are based on different methods of analysis (light scattering and diffraction, respectively), measures in different rheology regimes (static vs. dynamic), and may have different detection limits, differences in particles size may be explained.

The potentiometric titration results showed that the ferrihydrite surface is positively charged below the pH of 7.9 and negatively charged above it. The results are in good agreement with other literature findings that found values of pH at point of zero charge up to 8.2 [34,36,37,52,53]. Slightly higher values of the pH at point of zero charges for the ferrihydrite were measured when varying the electrolyte type and could be explained by the formation of asymmetrical ion paring [35]. This information will help us to understand the uptake process of various elements as a function of pH and their chemistry in solution as a function of pH. In addition, our novel approach investigating the effect of dissolved CO_2_ on surface properties of the ferrihydrite showed that dissolved CO_2_ can affect surface properties in the pH interval of 6–9, possibly forming carbonate species at ferrihydrite surface sites. This was the first experimental investigation that directly measured the effect of dissolved CO_2_ on the ferrihydrite surface. Our experimental result supports the computational outputs on carbonate adsorption onto ferrihydrite, which showed that carbonate ions affect surface coverage at pH interval of 4 to 9 [28]. A recent study that had indirectly measured the interaction of carbonates ions with the surfaces of ferrihydrite in batch experiments under a wide range of chemical conditions (pH, IS, CO_3_, and PO_4_ concentrations), and in the presence of competitive PO_4_, found that carbonates ions were adsorbed onto nanoparticles surface at pH 5–9, with a maximum at pH 6.5_._ Additionally, other surface complexation computational studies indicated that adsorbed species could be the following: bidentate inner sphere species (i.e., (≡FeO)_2_CO and (≡FeO)_2_CO···Na) and monodentate species (i.e., ≡FeOCO_2_) [54]. Their findings support our results and confirm the carbonates effect on iron oxides surface. Moreover, the pKa value determined from the second derivative of our potentiometric data showed a value of 6.23 which may be assigned to a cis isomer of –CO_2_H [38,39,40]. As the effect of carbonate investigated in our study has a wide perspective in natural and engineered environments, by its interference in many geochemical processes and settings, and its influence on elements uptake, more studies are suggested to be carried out to elucidate this mechanism and quantitatively estimate this effect.

Variations of surface area found in the literature are attributed to differences in the synthesis protocol as well post synthesis protocols such as washing, dispersion, thermal treatments, aging time at various pH values, etc. [35]. Similar values of surface area to ours were found in literature: 206 m^2^ g^−1^ [55], 200 m^2^ g^−1^ [56] and 245 ± 10 m^2^ g^−1^ [50]. This indicates similarities in the synthesis protocol and conditions. However, our experience and the literature studies have shown that even when the same synthesis method is followed, minor to large differences (±50 m^2^ g^−1^) in the surface area has been measured. This may be due to experimental artefacts such as the rapidity of iron solution titration, researcher experience and manual handling, the washing method post synthesis, the number of washings after synthesis, storage/aging method and time, aging pH, etc. Gustafsson (2003), as well as Cornell and Schwertmann (2003), mentioned that even if the same synthesis method is used ferrihydrite surface areas can vary between 200 and 320 m^2^g^−1^ and that this variation may also be due to the outgassing conditions during BET measurements or handling experiences [1,13].

From a literature comparison, it is to be noted that both the surface area as well as pH at point of zero charge of the ferrihydrite can vary among fresh, wet and dried nanoparticles [6,35,57].

### 4.2. Adsorption Studies

Limited adsorption studies on molybdenum [13] and vanadium [58] onto ferrihydrite are available in the literature, yet the available studies were carried out under different conditions (Table S3). Briefly, Gustafsson (2003) found similar trends of molybdenum adsorption onto ferrihydrite within the pH interval of 3 to 9. Using molybdenum concentration of 50 µmol L^−1^ and ferrihydrite concentration of 1 g L^−1^ they obtained 95–100% removal of molybdenum below pH 6.5; 68% at pH 7; ~15% at pH 8 and ~1.5% pH 9. Trefry and Metz (1989) showed that 80% of the vanadium was removed within two minutes from synthetic seawater (pH 8 and ionic strengths 0.7) spiked with 200 µmol L^−1^ vanadium, in the presence of 2 g L^−1^ ferrihydrite. A comparison of their results with ours shows that vanadium is rapidly taken up by ferrihydrite both in distilled water at pH 7 (this study) and in seawater at pH 8 [58].

For molybdenum, the results of particles concentration effect showed a general decreasing trend of the uptake capacity with increasing particles concentration, suggesting an under-saturation scenario at chosen anions concentration (i.e., 100 µmol L^−1^) and particles concentration below 1 g L^−1^. On the contrary, for the vanadium system, higher uptake capacities (ca 1.02 mmol g^−1^) were reached for all particles concentrations chosen.

The DLS results, which showed a decrease in particles size (implicitly higher surface area) and particles polydispersity with increasing particles concentration, can be related with and can support the adsorption trends seen in particles concentration effect studies, namely, the general increasing trend of the uptake capacities with increasing particles concentration, hence leading to higher adsorption performance. Additionally, the adsorption kinetic fits empirically suggested that at low particle concentrations (possibly also related to particles monodispersity/narrow particles size interval), the adsorption becomes preponderantly chemical, with anion binding occurring via strong chemical bonding, whereas at higher particles concentration (possibly also related to particles polydispersity/larger particles size interval) adsorption becomes predominantly physical, with anion binding occurring by wick bonds such as hydrogen and Van de Walls.

Hartzog et al. (2009) described the best protocol to compare, normalize and scale pH which depended on adsorption data [16]. They carried out adsorption experiments of As onto goethite-covered sand. By varying individual parameters such as absolute adsorbent and/or adsorbent concentration and relative adsorbate concentration in an arsenate goethite batch adsorption system at pH between 3 and 10, they found that the log Kd (distribution coefficient) approach is the most sensitive measure for adsorption studies compared to q and E. Their results, in association with other literature findings, suggested that normalizing the adsorption data to surface area rather than adsorbent mass reduces the variability in Kd and q for adsorption [16,59]. Thus, our uptake capacity data along with other literature findings were normalized per surface area and per g Fe for further references and were compared (Supplementary Table S4). As an approximated comparison with other anions’ adsorption onto ferrihydrite, one can be made by accounting for a study by Sannino et al. from which the Langmuir maximum uptake capacities of As (V) and Cr (VI) onto ferrihydrite, were 0.743 mmol As g^−1^ and 0.254 mmol Cr g^−1^, respectively [60]. The values are in the same order of magnitude as those from the current study. The small difference might be explained by lower pH (i.e., 4) compared to the present study (i.e., 7), and/or also by the chemistry of As and Cr in solution (that differ from Mo and V). A recent study on As (III) and As (V) adsorption by ferrihydrite, led to quantitatively ca. 5 times higher uptake capacities (5.4 mmol g^−1^ (Fe) and 5 mmol g^−1^ (Fe), respectively) at pH 7, indicating higher affinities of As species compared to Mo (0.986 mmol g^−1^ (Fe)) and V (1.33 mmol g^−1^ (Fe)) species at ferrihydrite surface sites. Additionally, As adsorption depends on As oxidation state: thus, in the pH interval 4 to 11 the As(III) adsorption increases with increasing pH and the As (V) adsorption decreases with increasing pH [53]. The same group found similar pH–adsorption performance trend for the W(VI) as for the As (V). Quantitatively they obtained maximum uptake capacity of 1.847 mmol W g^−1^ ferryhydrite, indicating a slightly higher affinity of W as compared to Mo (0.43 mmol g^−1^ ferryhydrite) and V (1.28 mmol g^−1^ ferryhydrite) [52]. More comparisons are given in Tables S3 and S4. Concluding, from the maximum uptake capacities comparisons of the ferrihydrite with other iron oxides for molybdenum and vanadium as well as other anions, it can be noted that ferrihydrite has superior sorption properties for molybdenum and vanadium. This enhances the importance and use of adsorption process in engineered settings (wastewater depollution, soil and sediments treatment), as well as its implications in natural environments (i.e., elemental cycling). 

The Kd values calculated for the current experiments at different particles concentrations, varied extensively for the molybdenum systems, while for the vanadium systems they were found in the same order of magnitude and decrease with increasing particles concentration (solid to solution ratio). Unfortunately, no coherent correlations of Kd with particles size, q or particles concentration could be made for the molybdenum system. On the other hand, the Kd values calculated for the current experiments at different anions concentrations have a similar trend for both, vanadium and molybdenum: namely, decreasing with increasing anion concentration and q values.

In theory, the Kd value should not vary with respect to the ratio of solid to the solution used. This is because, by definition, the Kd (mL g^−1^) is a normalized expression of the ratio of the ions adsorbed to the solid to the ions concentration left in the solution. However, in many studies has been observed that experimentally derived Kd values often exhibit a similar dependence to ours with respect to the ratio of solid to the solution used in the measurements: namely, Kd decreases as the solid-to-solution ratio increases [14,15]. Among the explanations found in the literature for this one would consider to be some experimental artefacts (i.e., high solids-content slurries can lead to less efficient separation of the solid phase, or heterogeneity the aqueous solution that are caused by mass transfer from the larger quantity of solids). Second type of explanation consists of particle –particle interactions that cause particles concentration effect. Particle –particles interactions, especially in systems with higher solid content can be regarded as potential physical blocking of some adsorption sites leading to a decrease of adsorption. In addition, also at high solid content, particle coagulation and flocculation may occur, leading to a decrease in particles surface area, hence less adsorption takes place. Accounting for the above, when a high solid content is used, the possibility that the measured Kd value to be underestimated may occur. On the other hand, measurements of Kd values conducted with a low solid-to-solution ratio would overestimate the magnitude of contaminant sorption (hence underestimate the extent of contaminant migration, for example) [14]. Concluding, explanations for Kd variations with particles concentration are still under debate and at some places are rather confusing. Thus, more experiments are suggested to be run under a lower and larger solid to solution ratio intervals. Additionally, to avoid Kd variability matters as mentioned above, it is advisable that, for a chosen case, the measurements of the Kd to be carried out under environmental conditions as close as possible to the ones that feature in the case under assessment. Alternatively, current data can be used in modelling codes which can calculate the estimated range of Kd values (as mean, min and max) for further use in models for anions migration in specific water sediments/soil with high content of iron oxihydroxides [15].

### 4.3. Simulation Outputs

The 3D plots of the inter-correlation between pH, particles concentration, and anions concentration offer a wider view on how multiple parameters affect simultaneously adsorption performance and are crucial information for process optimization and scaling up.

Thus, the molybdenum and vanadium uptake increase with increasing pH and particles concentration as well as anions concentration in solution, up to surface saturation conditions. A distinct profile was observed for vanadium compared to molybdenum in terms of the synergetic effect of pH and particle concentration, confirming the high affinity of vanadium for ferrihydrite surface sites over a larger pH interval, and additionally, with increasing particles concentration. When particles concentration and anions concentration dependences were plotted against anions uptake capacities, the 3D profiles were similar, however, the maximum uptake capacity for vanadium uptake was substantially bigger than for molybdenum. Quantitatively, the simulated maximum uptake capacities of both molybdenum and vanadium are an order of magnitude higher than those that were experimentally derived. Which should we trust? The use of simulated values for q in wastewater, soil and sediments applications offers significant guidance for depollution performance and also will adjust the amount of adsorbent necessary to achieve the required pollutant removal/immobilization. However, the experimentally derived values are the most realistic, and therefore it is essential to calculate correction factors for application to the simulated values when experimental data are not available. Moreover, it is suggested that more studies to be considered, in the future, in order to estimate the effect of other competitive ions and organic matter and derive correction factors to be applied from these respects. Examples of how these important correlations can be used in wastewater treatments applications from process engineering points of views, as well as may have implications in estimating contributions to elements geochemical cycling at specific geological sites, are given briefly below.

### 4.4. Implications and Applications of the Results

In the context of environmental protection, the concentration of pollutants released into surface waters from a polluted influent should respect strict environmental standards [3,4]. For this, wastewater treatments protocols, including adsorption based ones, are designed. Furthermore, for adsorption-based technologies, the depollution efficiency is a function of the amount of the adsorbent dosed in the wastewater treatment reactor/plant [61]. Thus, from a process engineer`s point of view, the maximum uptake capacity of any adsorbent used for specific pollutants is important and should be known, as well as its optimal working conditions. The 3D diagrams offer inter-correlations of these critical parameters for the ferrihydrite as a potential adsorbent, which can be used in batch or in column-based reactors, for molybdenum and vanadium removal from polluted wastewaters. Our simulation results can be used in a plethora of environmental scenarios to totally or partially reduce (down to the accepted/requested discharged limits) the concentration of molybdenum and vanadium from polluted effluents. For example, the decrease of molybdenum and/or vanadium concentration in some effluents from various industries (i.e., mining, battery, electroplating, etc.) can help to meet the local environmental standards for discharge; the decontamination of drinking water using the adsorption process as a tertiary treatment stage to achieve the limits set by environmental drinking water standards; the use of this process for water depollution and sediments treatment affected by accidental releases (i.e., red mud spill in Ajka, Hungary 2010 [62]).

We will briefly discuss one such example. Vanadium concentration in acidic wastewater may vary from 30–100 mg L^−1^ (0.6–2 mmol L^−1^) [63]. To consider the standards applicable for coal mines, the requirements of the general standards for discharge of environmental pollutants were set for vanadium at a maximum concentration of 0.2 mg L^−1^ (=0.004 mmol L^−1^), which was permitted to be discharged in inland surface water, public sewers, and marine or coastal areas [64]. With these figures in mind, at least ca 0.596 mmol vanadium per litre should be taken up to meet the environmental discharge standards. To do so, if an adsorption process is chosen for an effluent depollution with the ferrihydrite as a chosen adsorbent, then according to the current experimental vs. simulation results on ferrihydrite maximum uptake capacity for vanadium, ca. 0.003 g ferrihydrite per each litre of effluent should be considered according to simulation results (i.e., maximum uptake capacity derived from the simulation as ca 20 mmol V g ^−1^ FHY), as opposed to 0.465 g FHY per each litre of effluent that should be dosed if the experimental maximum uptake capacity (of 1.28 mmol V g^−1^ FHY) is taken into account. These calculations are very important guiding sources to maximize the depollution efficiency. In practice, they should be thought of in conjunction with other engineering and designing factors, such as rheology, mixing, reactor times, effluent flow, contact time, the competitive effect of other ions in effluent, etc. Similar examples can be calculated for molybdenum polluted wastewaters; however, extensive interpretation of similar approaches is beyond the purpose of this work, which aimed to offer only a brief insight. 

Another possible application is estimating the effect of ferrihydrite as a pool for molybdenum and vanadium uptake with implications on the geochemical cycling of elements. Thus, at deep-sea hydrothermal settings, where adsorption processes occur as ferrihydrite forms abundantly, if the variations of ferrihydrite input into seawater from a hydrothermal vent and the molybdenum or vanadium concentration in the surrounding seawater are known, these data can be used (via interpolation or extrapolation) to predict the amount of anions that can be scavenged from the seawater column along a deep-sea hydrothermal plume. Specifically, the continuous uptake of anions from the seawater reservoir via adsorption onto particles from the plume, assuming a molybdenum concentration in the seawater of 10 μg L^−1^ [65] and a vanadium concentration of ~2–3 µg L^−1^ [66] and taken an arbitrarily value of 1.5 g L^−1^ of ferrihydrite input into the seawater, then by interpolating on the appropriated plots and by taking into account the influence of the matrix (add 22% for molybdenum and 33% for vanadium [12]), we estimated 0.0183 mmol molybdenum and 0.0065 mmol vanadium per g of ferrihydrite can be removed by this process. This may explain why the molybdenum and vanadium concentration in the sediments near the vent is a few orders of magnitude higher than in surrounding seawater [58,67,68,69,70,71].

## 5. Conclusions

The molybdenum and vanadium adsorption experiments complemented by computational investigations in this study, have important implications on quantifying the uptake processes which take place in real, natural environments and engineered settings, including the ones controlling the availability of these micro-nutrients or pollutants (if concentrations exceed environmentally concerned values). Thus, the 3D inter-correlation plots of the main parameters that influence anions uptake onto ferrihydrite help to transpose the laboratory results to real ecosystems efficiently and economically.

## Figures and Tables

**Table 1:** Summary of the pseudo-second-order kinetic model parameters, calculated surface sites average, theoretic surface coverage, theoretic and practical/experimental percentages adsorbed and calculated distribution coefficients of molybdenum and vanadium adsorption onto ferrihydrite at three different particles concentrations.

System	Surface Sites Available (Mols Site L^−1^)	Th. Surf. Coverage	% th ads	% pr ads	q th (mmol g^−1^ FHY)	q pr (mmol g^−1^ FHY)	Pr R^2^	Calculated Kd (L g^−1^)
V-Cp = 0.1 g L^−1^	7.54 × 10^−5^	100%	75%	100%	0.75	0.98 ± 0.001	0.999	749.323
V-Cp = 1 g L^−1^	7.54 × 10^−4^	13%	100%	100%	0.1	0.974 ± 0.0019	0.999	453.820
V-Cp = 2 g L^−1^	15.08 × 10^−4^	7%	100%	100%	0.05	0.98 ± 0.00007	1	443.667
Mo-Cp = 0.1 g L^−1^	7.54 × 10^−5^	100%	75%	40%	0.750	0.468 ± 0.035	0.883	9.107
Mo-Cp = 1 g L^−1^	7.54 × 10^−4^	13%	100%	100%	0.1	0.0994 ± 0.00002	1	2983.375
Mo-Cp = 2 g L^−1^	15.08 × 10^−4^	7%	100%	100%	0.05	0.053 ± 0.00002	0.999	634.735

**Table 2:** Calculated R_L_ and Kd values for adsorption experiments run at varying anion concentrations and ferrihydrite concentration of 0.1 g L^−1^.

Mo/V Initial Concentration	RL		Kd (L g^−1^)	
	V	Mo	V	Mo
1	0.720	1.000	236.775	539.686
10	0.205	1.000	71.983	380.90
100	0.025	0.999	55.956	4.925
250	0.010	0.997	7.377	1.265
500	0.005	0.993	2.660	0.974
750	0.003	0.990	2.161	0.871

## Data Availability

Not applicable.

## Supplementary Materials

The following are available online at https://www.mdpi.com/article/10.3390/nano12030304/s1, Table S1: Kinetic modelling parameters for the molybdenum and vanadium adsorption at ferrihydrite particles concentrations from 0.1–2 gL^−1^, Table S2. Summary of adsorption isotherms parameters of molybdenum and vanadium at pH 7, Ionic strength (IS) 0.01, C_FHY_ = 1 g L^−1^, CMo/V= 1–750 μmol L^−1^, Table S3: Molybdenum and vanadium adsorption studies–literature comparative view, Table S4. Literature comparison of anions adsorption onto iron oxides.
