# Supplementary material for: Surface Coverage Simulation and 3D Plotting of Main Process Parameters for Molybdenum and Vanadium Adsorption onto Ferrihydrite
Source: Nanomaterials (Basel). 2022 Jan 18;12(3):304. doi: 10.3390/nano12030304 (PMC8840063; doi:10.3390/nano12030304)
Supplement: Supplementary file 1 [file nanomaterials-12-00304-s001.zip › nanomaterials-1523398-supplementary.pdf]

nanomaterials

Supplementary information

Surface Coverage Simulation and 3D Plotting of Main Process Parameters for Molybdenum and Vanadium Adsorption onto Ferrihydrite

Loredana Brinza

Department of Exact and Natural Sciences, Institute of Interdisciplinary Research, "Alexandru Ioan Cuza" University of Iasi, 700107 Iasi, Romania; Loredana.brinza@uaic.ro

Citation: Brinza, L. Surface Coverage Simulation and 3D Plotting of Main Process Parameters for Molybdenum and Vanadium Adsorption onto Ferrihydrite. *Nanomaterials* **2022**, *12*, 304. https://doi.org/10.3390/nano12030304

Academic Editor: Damien Faivre

Received: 8 December 2021
Accepted: 12 January 2022
Published: 18 January 2022

Publisher's Note: MDPI stays neutral with regard to jurisdictional claims in published maps and institutional affiliations.

Copyright: © 2022 by the author. Licensee MDPI, Basel, Switzerland. This article is an open access article distributed under the terms and conditions of the Creative Commons Attribution (CC BY) license (https://creativecommons.org/licenses/by/4.0/).

Material and methods

Apparatus and settings

A PHILIPS PW 1730/10 X-Ray powder diffractometer was used to confirm particles mineralogy and purity. Dried samples (overnight in the oven at 40ºC) were deposited onto a silicon sample holder and a diffraction pattern was collected using a Cu Kα radiation source with a wavelength of 1.54 Å. Data were collected for 2-theta between 5–70º at a scan rate of 0.08 °/minute; a step size of 0.01º, and working at 40 kV and 30 mA. These settings lead to long scans (~14 h) which insured that the presence of even small quantities of other phases could be identified. Scans of the experimental materials were compared with standard traces from the d JCDD database.

Surface area measurements of the ferrihydrite were performed following the standard protocol of Micrometrics Gemini V – based on Brunauer, Emmett, and Teller (BET) theory. Micromeritics Gemini V Series analyser was used to measure ferrihydrite surface area using He gas. A known amount of freeze-dried ferrihydrite was degassed under N_2 with a VacPrep degasser for 12 hours and the surface area was determined at a fixed gas (He) evacuation rate of 100 mmHg min^{-1}, an optimum pressure of 765 mmHg, and an equilibration time of 5 sec.

Potentiometric itrations were conducted in 250 mL beakers on a magnetic stirrer plate (600 rpm). Before each titration, the pH electrode was manually calibrated using a VWR certified pH standard solution. The working temperature was room temperature (21 ± 0.5 °C). A standard titration protocol involved equilibrating an ferrihydrite suspension in 100 mL of electrolyte background solution (NaCl, 0.1 mol L^{-1}), then titrating both from high to low pH and from low to high pH with either HCl or NaOH (0.1 mol L^{-1}) to establish hysteresis plots.

The ferrihydrite aggregate size in solution was measured in a static regime with a Dynamic Light Scattering device (Zetasizer Nano ZS) from Malvern Instruments. Ferrihydrite particle size measurements were performed at particles concentrations of 0.001; 0.002; 0.004 and 0.006 g L^{-1}, in water and at pH 7. The measurements were performed in 1 cm plastic cuvettes at 21 °C using a laser beam with a λ= 633 nm at an angle of the detector of 173º.

Adsorption modelling

Kinetic modelling:

The uptake capacities at equilibrium for the experimental data at varying particles concentration were calculated by fitting the experimentally derived uptake capacities from the adsorption experiments where particles concentration was varied, to empirical pseudo-first and pseudo-second-order models [1–3] shown below:

Pseudo first order model:

Non linear form: $\frac{dq_t}{dt} = k_1 \times (q_e - q_t)$ (Eq. S1)

Linear form: $\ln(q_e - q_t) = \ln q_e - k_1 \times t$ (Eq. S2)

$k_1 = pseudo\ first\ order\ kinetic\ rate\ cofficient, min^{-1}$

Pseudo second order model:

Non linear form: $\frac{dq_t}{dt} = k_2 \times (q_e - q_t)^2$ (Eq. S3)

Linear form: $\frac{1}{q_t} = \frac{1}{k_2 \times q_e{}^2} + \frac{1}{q_e} \times \mathrm{t}$ (Eq. S4)

$k_2 = pseudo\ second\ order\ kinetic\ rate\ cofficient, g\ mmol^{-1} min^{-1}$

Where k is the rate constant of adsorption, (min^{-1} for the PFO and g $mmol^{-1}$ min^{-1} for PSO); q_e is the amount of metal adsorbed at equilibrium, (mmol g^{-1}) and qt is the amount of metal adsorbed per g ferrihydrite, (mmol g^{-1}) at any time, t, in min.

The PFO kinetic model implies mainly a physical adsorption mechanism based on weak interactions (e.g., hydrogen bonding or Van der Walls forces) and the metal sorption rate is proportional to the number of vacant sites at bio-sorbent surface. The PSO kinetic model will indicate the chemical sorption mechanism assuming strong covalent bonding and that the rate of metal sorption is proportional to the square of the number of vacant sites at sorbent surface.

Results

Kinetic modelling

Table S1. Summary of the kinetic modelling parameters.

System	Parameters and statistics	PFO model	PSO model
V, Cp 0.1 g L^{-1}		$q_e = 0.97 \pm 0.003\ mmol\ g^{-1}$<br>$k_1 = 0.30 \pm 0.022\ min^{-1}$	$q_e = 0.98 \pm 0.001\ mmol\ g^{-1}$<br>$k_2 = 1.67 \pm 0.08\ g\ mmol^{-1}\ min^{-1}$
	Red. X^2	0.0001	$5.61 \cdot 10^{-6}$
	Adj. R^2	0.9990	0.9999
V, Cp 1 g L^{-1}		$q_e = 0.974 \pm 0.0019\ mmol\ g^{-1}$<br>$k_1 = 0.636 \pm 0.32\ min^{-1}$	$q_e = 0.973 \pm 0.0049\ mmol\ g^{-1}$<br>$k_2 = 5.065 \pm 3.24\ g\ mmol^{-1}\ min^{-1}$
	Red. X^2	2.53×10^{-5}	1.23×10^{-4}
	Adj. R^2	0.9997	0.998
V, Cp 2 g L^{-1}		$q_e = 0.98 \pm 0.00007\ mmol\ g^{-1}$<br>$k_1 = 0.715 \pm 0.0276\ min^{-1}$	$q_e = 0.981 \pm 0.001\ mmol\ g^{-1}$<br>$k_2 = 9.294 \pm 4.3\ g\ mmol^{-1}\ min^{-1}$
	Red. X^2	3.74×10^{-8}	1.97×10^{-5}
	Adj. R^2	1	0.9999
Mo, Cp 0.1 g L^{-1}		$q_e = 0.425 \pm 0.039\ mmol\ g^{-1}$<br>$k_1 = 0.047 \pm 0.018\ min^{-1}$	$q_e = 0.468 \pm 0.035\ mmol\ g^{-1}$<br>$k_2 = 0.134 \pm 0.057\ g\ mmol^{-1}\ min^{-1}$

	Red. X^2	0.00596	0.0029
	Adj. R^2	0.768	0.883
Mo, Cp 1 g L^{-1}		$q_e = 0.0994 \pm 0.00002\ mmol\ g^{-1}$<br>$k_1 = 0.52021 \pm 0.014\ min^{-1}$	$q_e = 0.0995 \pm 0.00001\ mmol\ g^{-1}$<br>$k_2 = 21.12 \pm 10.66\ g\ mmol^{-1}\ min^{-1}$
	Red. X^2	4.90×10^{-9}	3.65×10^{-6}
	Adj. R^2	1	0.997
Mo, Cp 2 g L^{-1}		$q_e = 0.053 \pm 0.00002\ mmol\ g^{-1}$<br>$k_1 = 0.668 \pm 0.092\ min^{-1}$	$q_e = 0.05336 \pm 0.00001\ mmol\ g^{-1}$<br>$k_2 = 37.60 \pm 0.8494\ g\ mmol^{-1}\ min^{-1}$
	Red. X^2	3.14×10^{-9}	1.41×10^{-6}
	Adj. R^2	0.9999	0.959

Adsorption isotherms modelling

Fitting the molybdenum and vanadium adsorption experimental data at varying anions concentration with the Langmuir and Freundlich Isotherm Models, a better regression factor by Langmuir isotherm was calculated. According to the Langmuir model hypothesis and assumptions, empirically it can be suggested that the adsorption of both molybdenum and vanadium takes place preponderantly as a monolayer; all adsorption sites being energetically equal homogenously spread over the substrate surface.

Table S2. Summary of the adsorption isotherms modelling parameters.

	**Langmuir isotherm**		**Freundlich isotherm**	
	**Mo**	**V**	**Mo**	**V**
Parameters	$q_{max} = 0.431 \pm 0.047$ mmol g^{-1}<br>b = 936	$q_{max} = 1.28 \pm 0.29$ mmol g^{-1}<br>b = 14.69	$K_f = 0.676 \pm 0.150$ mmol g^{-1}<br>n = 0.36 ± 0.039	$K_f = 1.904 \pm 0.5106$ mmol g^{-1}<br>n = 0.51 ± 0.046
Statistics	Adj. $R^2 = 0.949$<br>Red. $X^2 = 18.44$	Adj. $R^2 = 0.920$<br>Red. $X^2 = 30.07$	Adj. $R^2 = 0.828$<br>Red. $X^2 = 62.73$	Adj. $R^2 = 0.785$<br>Red. $X^2 = 76.82$

Maximum loading capacities or maximum uptake capacities given by a Langmuir isotherm allow the comparison with different sorbents to remove and control the availability of molybdenum and vanadium. It is important to note that such comparisons are valid only if specified process conditions are similar. A brief comparison with the literature data is offered in the discussion section below.

Literature comparison of the adsorption results

Table S3. Molybdenum and vanadium adsorption studies – literature comparative view.

**System**	**Conditions**	**Results (sorption efficiency)**	**Reference**
Mo adsorption onto FHY	C_{Mo} = *50* µmol L^{-1}; C_{FHY} =1g L^{-1} ; *pH 3–8*	pH below 6.5: 95–100%; pH 7: 68 %; pH 8 ~ 15 %; and pH 9 ~ 1.5%	[4]
	C_{Mo} = *100* µmol L^{-1}; C_{FHY} = 0.1g L^{-1} ; *pH 4–9*; IS=0.01	pH below 6: 90–100%; pH 7 ~ 60 %; pH 8 ~ 20 % and pH 9 ~ 3%	This study
V adsorption onto FHY	C_V = 200 µmol L^{-1} V, C_{FHY} =2g·L^{-1} , pH 8, IS= *0.7(seawater)*	>80 %	[5]
	C_V = *100* µmol L^{-1}; C_{FHY} = 0.1g L^{-1} ; pH = 7; IS=*0.01*	>90 %,	This study

Table S4. Literature comparison of anions adsorption onto iron oxides.

Anion	Iron oxides - adsorbent and conditions	q (mmol/gFe)	q (mmol/m²)	References
V	FHY – slurry; SA=200m²·g⁻¹; pH 7; Ci = 1-750 µmol L⁻¹;	1.333 mmol g⁻¹ Fe (from 1.28 mmol g⁻¹FHY)	0.0049*	This study
Mo	FHY – slurry; SA=200m²·g⁻¹ ; pH 7; Ci =1- 750 µmol L⁻¹;	0.986 mmol g⁻¹Fe (from 0.43 mmol g⁻¹FHY)	0.0027*	This study
P	Biogenic oxides; pH 6.4; Ci = 100 µmol L⁻¹;	1.77	n.a.	[6]
As	Goethite coated sand; SA=178m²·g⁻¹; pH 7; Ci = 67 µmol L⁻¹	0.134	2.82*10⁻⁶ §	[7]
As	Goethite; SA= 20m²·g⁻¹; pH 7; Ci = 100 µM; Cp=0.9g·L⁻¹	0.0760	0.0024	[8]
P	FHY slurry; SA=200-320m²·g⁻¹; pH 7.18; Ci=200 µmol L⁻¹	0.182	0.0039	[4]
W	FHY slurry; SA=200-320m²·g⁻¹; pH 7.29; Ci=50 µmol L⁻¹	0.086	0.0019‡	[4]
Mo	FHY slurry; SA=200-320m²·g⁻¹; pH 7.09; Ci=50 µmol L⁻¹	0.062	0.0021‡	[4]

*conversion to mmolg⁻¹ Fe was made taking into account that 1 g ferrihydrite contains 600 mg of Fe (measured value);.
§ for comparison a synthesized goethite coated sand with SA = 178 m²·g⁻¹ and 6.83 10⁻⁵ mol Fe g⁻¹ material was used.
‡An averaged surface area of 260 m²·g⁻¹ and iron content per g ferrihydrite of 558 mg Fe g⁻¹ ferrihydrite was used.

References

1. Lagergren, S. Zur theorie der sogenannten adsorption gelöster stoffe, Kungliga Svenska Vetenskapsakademiens. *Handlingar* **1886**, *24*, 1–39.
2. Ho, Y.S. and McKay, G. A Comparison of Chemisorption Kinetic Models Applied to Pollutant Removal on Various Sorbents. *Process Saf. Environ. Prot.* **1998**, *76*, 332–340.
3. Ho, Y.-S. Review of second-order models for adsorption systems. *J. Hazard. Mater.* **2006**, *136*, 681–689.
4. Gustafsson, J.P. Modelling molybdate and tungstate adsorption to ferrihydrite. *Chem. Geol.* **2003**, *200*, 105–115.
5. Trefry, J.H. and Metz, S. Role of hydrothermal precipitates in the geochemical cycling of vanadium. *Lett. Nat.* **1989**, *432*, 531–534.
6. Rentz, J.A.; Turner, I.P. and Ullman, J.L. Removal of phosphorus from solution using biogenic iron oxides. *Water Res.* **2009**, *43*, 2029–2035.
7. Hartzog, O.K.; Loganathan, V.A.; Kanel, S.R.; Jeppu, G.P.; Barnett, M.O. Normalization, comparison, and scaling of adsorption data: arsenate and goethite. *J Colloid Interface Sci.* **2009**, *333*, 6–13.
8. Kersten, M. and Vlasova, N. Arsenite adsorption on goethite at elevated temperatures. *Appl. Geochem.* **2009**, *24*, 32–43.
